# Heart rate variability in children with tricyclic antidepressant intoxication

**DOI:** 10.1186/cc12208

**Published:** 2013-03-19

**Authors:** EC Dinleyici, Z Kilic, S Sahin, R Tutuncu-Toker, M Eren, ZA Yargic, P Kosger, B Ucar

**Affiliations:** 1 Eskisehir Osmangazi University Faculty of Medicine, Eskisehir, Turkey

## Introduction

Tricyclic antidepressant (TCA) intoxication is among the most common causes of poisonings in our country. The TCA group of drugs affects the central nervous and cardiovascular systems, resulting in severe arrhythmia and death. Heart rate variability (HRV) analysis is a non-invasive assessment method that allows evaluation of the cardiac autonomic (sympathetic and parasympathetic) activity. The aim of this study was to evaluate HRV in children requiring ICU stay due to TCA poisoning.

## Methods

Twenty children with isolated TCA poisoning aged between 3 and 16 years who were hospitalized in the pediatric ICU, between March 2009 and July 2010, and 20 healthy children as a control group were enrolled. Clinical and electrocardiographic (ECG) findings were noted in the TCA poisoning group. In both groups, 24-hour time domain HRV analysis (SDNN, SDANN, SDNNi, RMSDD, NN50, and pNN50) was performed. We also recorded frequency domain analysis results at the first 5 minutes and the last 5 minutes of the 24-hour record (VLF, nLF, nHF, LF/HF ratio).

## Results

The average level of TCA in the study group was 1,116 ± 635 and TCA levels were positively correlated with the duration of QRS interval (*P <*0.01). In time-domain nonspectral evaluation, SDNN (*P <*0.001), SDNN (*P <*0.05), RMSDD (*P <*0.01), and pNN50 (*P <*0.01) were found significantly lower in the TCA intoxication group compared with the control group, while NN50 (*P <*0.01) was significantly higher in value. The spectral analysis (frequency domain) of data recorded at first 5 minutes after intensive care admission showed that the values of the nLF (*P <*0.05) and LF/HF ratio (*P *= 0.001) were significantly higher in the TCA intoxication group than the controls, while nHF (*P *= 0.001) values were significantly lower. The frequency domain spectral analysis of data recorded at the last 5 minutes showed a lower nHF (*P *= 0.001) in the TCA intoxication group than the controls, and the LF/HF ratio was significantly higher (*P <*0.05) in the intoxication group. SDNN (*P <*0.001), RMSDD (*P <*0.01), SDNNi (*P <*0.01), and pNN50 (*P <*0.01) levels were higher in patients with positive ECG findings than those without positive ECG findings. The LF/HF ratio was higher in seven children with seizures (*P <*0.001).

## Conclusion

Existing findings give us an idea about HRV's value to determine arrhythmia and predict convulsion risk in TCA poisonings. HRV can be used as a non-invasive method in determining the treatment and prognosis of TCA poisoning.

